# The Separation and Utilization of Biomass Components in the Pre-Hydrolysis Liquor of Kraft-Based Dissolving Pulp Production Process—A Review

**DOI:** 10.3390/polym18040463

**Published:** 2026-02-12

**Authors:** Zongquan Li, Yuhang Wang

**Affiliations:** State Key Laboratory of Green Papermaking and Resource Recycling, Qilu University of Technology (Shandong Academy of Sciences), Jinan 250353, China; 13279539167@163.com

**Keywords:** pre-hydrolysis liquor (PHL), Hemicelluloses, lignin, separation, utilization, furfural, acetic acid

## Abstract

The effective utilization of biomass components in the pre-hydrolysis liquor (PHL) of lignocellulose is a crucial way for traditional pulp and paper mills converting into biomass refining facilities. In the present work, separation technologies are summarized and reviewed—including acidification, ethanol precipitation, flocculation and coagulation, adsorption, solvent extraction, enzyme treatment, and oxidation—with regard to component separation and impurity removal. The utilization of hemicelluloses from PHL for the production of furfural, adhesive and biofuel, as well as the application of lignin separated from PHL and the full components utilization of PHL without separation is reviewed and analyzed.

## 1. Introduction

Dissolving pulp is an important raw material for the production of cellulose derivatives. Compared to conventional paper pulp, dissolving pulp requires higher cellulose contents and lower hemicellulose and lignin contents. Dissolving pulps are mainly produced through acid sulphite (AS) pulping processes and the pre-hydrolysis kraft (PHK) process [1]. The dissolving pulp produced via the latter process accounts for approximately 56% of global production [1]. In the PHK process, high-temperature extraction with water (or steam), also known as autohydrolysis, is used for pre-hydrolysis, as the acetyl groups in hemicelluloses tend to degrade and form acetic acid, which can catalyze the hydrolysis reactions. In addition, diluted acids, mainly sulfuric acid, are also used for the pre-hydrolysis of woodchips, but due to the disadvantages of the acid pre-hydrolysis approach, including undesirable corrosive effects, extensive lignin condensation, and undesirable cellulose hydrolysis, the autohydrolysis method is more commonly practiced for the production of dissolving pulp [2,3]. Both hardwoods and softwoods can be used in the PHK process, and the main hemicelluloses in hardwoods and softwoods are glucouronoxylan (Xylan) and galactoglucomannan (GGM), respectively [4,5]. Compared to softwoods, hardwoods are more appropriate for autohydrolysis due to their higher content of hemicelluloses and acetyl groups and their hemicellulose structure [6]. During pre-hydrolysis, most of the hemicellulose in the fiber material degrades and dissolves into a pre-hydrolysis liquor (PHL) (also known as hydrolysates), which mainly occurs in the oligomeric form [3,7]. Additionally, a small portion of lignin and degraded carbohydrate products, such as acetic acid, furfural, and 5-hydroxymethylfurfural (5-HMF), also dissolve into the PHL. The components of the PHL vary depending on the hydrolysis conditions and raw materials, and the hemicellulose sugar, lignin, and acetic acid contents are generally 30–60 g/L, 6–15 g/L, and 3–12 g/L, respectively, including a small amount of furfural [8,9,10,11,12,13]. The pre-hydrolysis liquor generated during the production of dissolving pulp can be co-combusted with the black liquor produced in kraft pulping, but this results in the waste of biomass resources. In recent years, many studies have been conducted on the utilization of biomass resources in PHL. The efficient utilization of the biomass components of PHL produced during the dissolving pulp production is one of the key pathways for converting traditional pulp and paper mills into the biomass refining facilities [14,15]. In this context, different biomass components can be separated and utilized individually, or relevant components can be directly utilized without the need for separation and purification. In order to gain a more comprehensive understanding of the utilization of biomass components in PHL, the separation and utilization of biomass components are reviewed.

## 2. Separation and Utilization of Hemicellulose Sugars in PHL

### 2.1. The Removal of the Impurities and the Separation of Hemicellulose Sugars in PHL

In the PHK process, the aims are to remove the hemicelluloses from the raw materials and to activate and increase the accessibility of the lignocellulosic material in the subsequent kraft pulping process [16]. During pre-hydrolysis, most of the hemicellulose sugars are released into the PHL, forming the primary component of the liquor. As an important biomass resource, they serve as key raw materials for the production of platform chemicals, materials, and bioenergy. To effectively utilize the hemicellulose sugars in PHL, it is essential to remove impurities—mainly lignin (including colloidal lignin and dissolved lignin). This is because, in addition to affecting the purity of hemicellulose sugars, lignin can cause membrane fouling during subsequent ultrafiltration or nanofiltration processes for concentration and removal of small molecular impurities, thereby reducing production efficiency and membrane decay [17,18]. When fermenting the sugars from PHL to produce biofuels, lignin-related impurities and degraded carbohydrate products, such as furfural and 5-hydroxymethylfurfural (5-HMF), also exhibit toxicity to fermentation strains [19,20].

Many researchers have investigated methods for removing lignin-dominated impurities from PHL and recovering hemicellulose sugars. For instance, an acid treatment can reduce lignin solubility, causing it to precipitate and be removed from the PHL [21,22]. However, acid treatments have a limited effectiveness for the removal of lignin and often result in significant sugar loss [23,24]. Ethanol treatments can directly precipitate hemicellulose sugars from PHL due to their insolubility in ethanol, but the yield and purity are relatively low [25]. It has been demonstrated that ethanol treatments of PHL cause the precipitation of hemicelluloses with a high molecular weight [26,27]. Both acidification and ethanol treatments can lead to the precipitation of lignin–carbohydrate complexes, which results in the loss of the hemicelluloses [24]. The results obtained by Shen et al. established that, compared to ethanol, acetone was more effective and selective for hemicellulose separation from the PHL [28]; however, acetone is more toxic.

Various adsorbents—including activated carbon (AC) [29,30,31,32,33,34,35,36], calcium hydroxide (lime) or calcium carbonate [13,32], zeolite [37], hydrotalcites [38], magnetite-adorned reduced graphene oxide [39], and ion-exchange resins [35,40,41]—have been used to adsorb and remove lignin and other impurities from PHL. Among these adsorbents, lime and activated carbon are commonly used in practice for the removal of impurities, mainly lignin, from PHL due to their low cost and high accessibility, but a higher amount of lime or activated carbon is required to achieve the desired removal effect, and the sugar loss is high due to their adsorption on the adsorbents [42,43]. For activated carbon, in addition to the high dosage requirement, there is a 10% loss caused by each thermal reactivation cycle during regeneration [44]. With the over-liming treatment, the impurity removal efficiency is limited, and it should be used in combination with other methods. Furthermore, the additional waste generated after the over-liming treatment will lead to environmental challenges [45]. It is believed that the combination of over-liming and activated carbon is an effective method for purity removal in PHL, as different impurities can be removed effectively in both treatment stages [46]. Ion-exchange resin adsorption is also a commonly used method for effectively removing impurities, such as lignin, from PHL. The efficiency of ion-exchange resin adsorption and the removal of impurities are related to the type of resin. It is believed that the polystyrene divinylbenzene copolymer resin can selectively adsorb lignin through π-π and van der Waals forces [47]. Chen et al. found that anion-exchange resins can adsorb organic acids and monomeric sugars, while cation-exchange resins can effectively adsorb phenolic compounds [41]. Yu et al. compared the performances of different exchange resins for the removal of lignin from PHL, and the results demonstrated that both Amberlite IRA-400 (OH^−^) and XAD-4 are effective for the removal of acid-soluble lignin, but the XAD-4 treatment caused a loss of 88% of xylo-saccharides as compared to 21% with the IRA-400 (OH^−^) treatment [48]. Another study also identified the poor selectivity of XAD-4 for lignin removal [34]. In addition, different sugars can be further separated using different types of ion-exchange resins due to their distinct absorption selectivity [49].

While using various adsorption methods to remove lignin, other impurities such as furfural and acetic acid are also removed. The impurity removal efficiency is dependent on the adsorbent dosage and treatment conditions [30]. At high adsorbent dosages, the impurity removal efficiency is generally higher, but this also leads to greater sugar loss, sometimes exceeding 30% [50]. The loss of hemicellulose sugars during the removal of lignin in PHL is related to the presence of lignin–carbohydrate complexes (LCCs). When the PHL is treated with xylanase, the sugar loss in the subsequent activated carbon adsorption stage is lower, which is due to the depolymerization of the poly-sugars in PHL as well as the removal of the lignin released from the LCCs through the xylanase treatment [30].

In addition to adsorbents, cationic or non-ionic flocculants or coagulants—such as cationic polyacrylamide (CPAM), poly dimethyl diallyl ammonium chloride (PDADMAC), polyethyleneimine(PEI), polyaluminum chloride (PAC), chitosan, polyethylene oxide (PEO), and other polymers—are also used to treat PHL. Lignin tends to form a composite with the polymers that are to be removed [51,52,53,54,55]. These flocculation methods are very effective in removing colloidal lignin, but the removal efficiency of the small-molecule dissolved lignin is limited.

Liquid–liquid extraction methods have been used to directly extract the impurities, mainly lignin, in PHL and recover hemicelluloses. Some solvents—such as ethyl acetate, 1-butanol, isobutyl acetate, methyl-isobutyl-ketone (MIBK), and n-hexane—have been evaluated for the impurity removal [56,57,58]. It has been established that the selectivity of the recovering hemicelluloses depends on the solvents and extraction conditions used [56]. Ethyl acetate extraction is believed to be an effective solvent for the extraction of phenolic compounds from PHL, improving the fermentation efficiency of hemicellulose sugars [58,59]. However, these liquid–liquid extraction methods suffer from a significant loss of hemicellulose sugars and a large degree of solvent recovery, resulting in low economic feasibility.

Biological enzymes, such as laccase, manganese peroxidase, and horseradish peroxidase, can catalyze the polymerization of lignin in PHL, resulting in an increase in their molecular weight and a reduction in solubility, leading to their precipitation and removal [60,61,62]. When cationic flocculants or coagulants are used to remove lignin from the PHL, the anionic polygalacturonic acid released during pre-hydrolysis affects the efficiency of the cationic polymers. The pectinase treatment can improve the efficiency of the cationic polymer for lignin removal due to the degradation of polygalacturonic acid to monomers [63].

In addition to traditional methods, such as adsorption, to remove impurities and obtain purified sugars, researchers have recently reported a new method for separating xylose from PHL. In this process, the PHL is added to a methanol solution of phenylboronic acid to generate boronic acid ester xylose. The produced boronic acid ester xylose is then dissolved in ethyl acetate and subjected to an ester exchange reaction with 1,2-propanediol, resulting in the precipitation of xylose. After filtration, pure xylose is obtained [64].

The lignin in PHL can also be removed via selective degradation. For example, gas-phase pulsed corona discharge was used to oxidize lignin in PHL to enhance ultrafiltration for the recovery of galactoglucomannan, and the results revealed that oxidation significantly improved the filterability of the oxidized PHL due to its decreased viscosity, although its effect on the fouling of the hydrophilic cellulose-based ultrafiltration (UF) membranes was slightly smaller [65]. Zhang et al. reported a novel method for PHL lignin removal via a treatment with BiOCl and BiOBr. It was believed that BiOCl and BiOBr not only removed the lignin via adsorption but also caused lignin degradation via photocatalysis [66]. The lignin removal efficiency of this method is limited, and it should be used in combination with other methods for high lignin removal efficiency.

In general, a single treatment stage has limited efficiency in removing lignin or other impurities. Therefore, when separating high-purity sugars from PHL, a combination of two or more methods is commonly used to improve the removal efficiency of lignin and other impurities. For example, Chen et al. treated the PHL of poplar with laccase and xylanase after neutralization with calcium hydroxide, combined with subsequent activated carbon adsorption, and prepared xylo-sugar powder after freeze-drying [8]. Shen et al. used activated carbon adsorption combined with ion-exchange resin adsorption to remove impurities, including lignin, in PHL and then used membrane filtration to concentrate and prepare a high-concentration (over 22%) hemicellulose sugar solution [67]. The efficiency of some typical methods for impurity removal is compared in Table 1.

When the combined processes are used for the separation and purification of the biomass compositions of PHL, more stages are used, and higher purity sugars may be obtained, but the yield of sugars tends to decrease, and the cost should also be considered in practice. When separating and purifying sugars from PHL, adopting a simple and effective process to efficiently remove impurities while reducing sugar loss is crucial.

### 2.2. The Utilization of the Hemicellulose Sugars in PHL

Hemicellulose sugars are the dominant components in PHL. Their concentration varies depending on the hydrolysis conditions and raw materials. After separation and purification, monomer sugars or oligo sugars can be obtained from PHL. Before separation and purification, an acid hydrolysis treatment can be used to obtain complete monomer sugars, such as xylose, for the pre-hydrolysis of hardwoods. The monosaccharides obtained through separation and purification, mainly xylose, can be used to produce xylitol via catalytic hydrogenation or fermentation and applied in food or pharmaceutical sectors [36], and the oligosaccharides from softwood PHL, mainly galactoglucomannan, and the xylo-oligosaccharide from hardwood PHL can also be applied in the same sectors [71,72]. The application of monosaccharides or oligosaccharides in the food and pharmaceutical sectors represents a traditional and well-known use of sugars, which will not be discussed in detail.

#### 2.2.1. Biofuel Production

Cellulose-based hydrolysis and fermentation for biofuel production is very popular and has been studied for many years, as well as the hemicellulose sugars from the lignocellulose [73,74]. The hemicellulose sugars in PHL can also be used as carbon sources for fermentation to produce biofuels such as ethanol, bio-oil, and biohydrogen. Bioethanol is a popular biofuel product obtained from PHL. In general, detoxification is required as PHL is used for biofuel production via fermentation. For example, Huang et al. treated the PHL of hardwood with acid to convert the xylo-oligosaccharides into xylose, and then a polystyrene divinylbenzene (PS-DVB) resin was used for the adsorption and removal of lignin and carbohydrate derivatives. After the resin adsorption, 97% of lignin, 92% of furfural, and 97% of 5-HMF were removed, and the xylose yield was as high as 96%. Compared with PHL without resin adsorption treatment, the ethanol yield produced with resin-absorption-treated PHL fermented with *Pichia stipites* increased by 162% and 282% for the PHL containing 30 g/L and 50 g/L of xylose, respectively [40]. Yu et al. also found that a strong anion-exchange resin containing tertiary amine functional groups (Amberlite IRA-400) was effective for detoxification as it removed aromatic-based inhibitors, including acid-soluble lignin in the PHL of polar, and the resin treatment of PHL significantly improved its fermentability. When the detoxified PHL was hydrolyzed with xylanase followed by fermentation with a genetically modified xylose-fermenting yeast strain of *Saccharomyces cerevisiae*, the produced ethanol titer attained was present at 41.5 g/L, with an ethanol yield and sugar efficiency of 89.6% and 95.3%, respectively [48]. In another study, methyl-isobutyl-ketone (MIBK) was used to extract the inhibitors in sugarcane bagasse hydrolysates, and this resulted in the removal of 69.0% of phenolics; 85.4% of acetic acid; and 100.0% of 5-HMF and furfural. The detoxified hydrolysate was fermented with *S. passalidarum* to produce ethanol with a yield and productivity of 75.6% and 0.41 g/g, respectively [57].

In addition to ethanol, a bioenergy product mixture of acetone−butanol−ethanol (ABE) can be obtained via the fermentation of hemicelluloses related sugars contained in PHL after detoxification via an activated charcoal treatment for the reduction in phenolic compounds. The fermentation of the PHL of poplar/pine with *Clostridium acetobutylicum ATCC 824* produced a solvent yield of 0.25/0.28 g of solvent/g of sugar, which demonstrated that it was technically feasible to utilize hemicellulose-rich PHL as the feedstock for the production of ABE [75]. Biodetoxification is also an alternative for ABE production via the fermentation of PHL. For example, Theiri et al. purposed a process that includes the detoxification of a wood PHL via a combined method of coagulation–flocculation followed by the co-culture of two bacteria (*U. thermosphaericus* and *C. taiwanensis*) and the simultaneous hydrolysis of the hemicelluloses with another bacterium (*P. campinasensis*), in which 56% of phenolic compounds were removed, and the butanol production via the fermentation of the detoxified PHL could reach up to 6.8 g/L [76].

In addition to separating sugars for the production of biofuels, PHL can also be used directly as a raw material for biofuel production. For example, it has been established that the PHL obtained from sugarcane bagasse through acid pre-hydrolysis can be directly fermented with *Lipomyces starkeyi DSM 70296* without separation to produce fatty acids, and the fatty acid composition produced by *Lipomyces starkeyi* is similar to that of vegetable oils, which demonstrates the potential of this fatty acid for biodiesel production. In addition, it has been established that furfural, 5-hydroxymethyl furfural (5-HMF), and acetic acid in the PHL had no inhibitory effects on fermentation [77].

Biohydrogen is another bioenergy product derived from hemicellulose-rich PHL. In a recent paper, hemicellulose-rich softwood PHL was fermented with optimized microorganisms. Before fermentation, the softwood PHL was freeze-dried and dissolved in water for fermentation, and the results revealed that the 100% PHL feed produced a biogas containing 31% of H_2_ with a rate of 497 mL H_2_/L/d. More than 80% of the monomeric and oligomeric sugars were metabolized. In addition, most of the fermentation inhibitors, such as 5-HMF, vanillin, and furfural, were degraded by the mixed microbial cultures during fermentation. The results demonstrated that the softwood hydrolysates could be used to supplement other readily fermentable feedstocks to improve biogas’s production potential [78].

When PHL is used to create bioenergy products through fermentation without inhibitor removal, the selection of bacterial strains is crucial. In addition, increasing the concentration of sugars in the PHL is important to improve the concentration of bioenergy products.

#### 2.2.2. Adhesive Production

**Figure 1:**
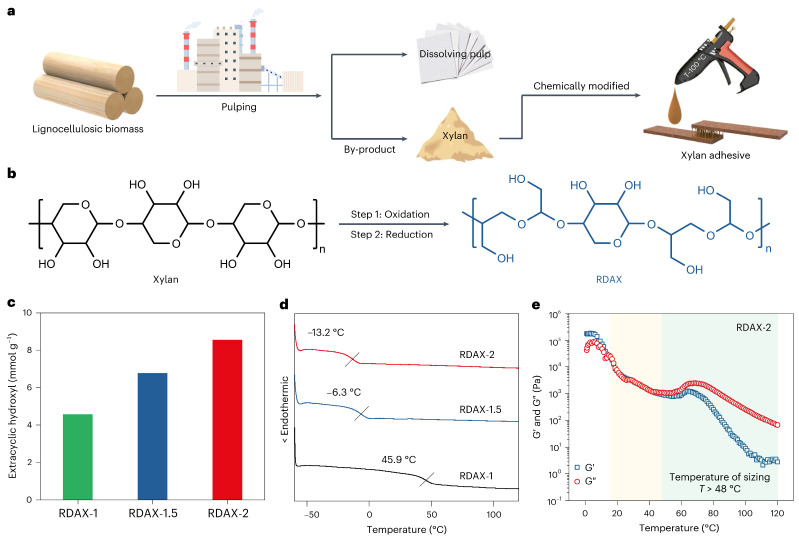
The preparation and characterization of the xylan adhesives. Reprinted from [79], Copyright (2025), with permission from Springer Nature. (**a**,**b**) The preparation of the xylan adhesives via a continuous redox reaction. (**c**) The extracyclic hydroxyl content of the xylan adhesives. (**d**) DSC curves of the xylan adhesives. The xylan adhesives exhibited different glass transition temperatures with different contents of extracyclic hydroxy groups. (**e**) Curves of RDAX-2 are divided into three regions: the white area marks the failure zone, the yellow area denotes the functional zone, and the green area denotes the sizing zone. For RDAX-1, RDAX-1.5, and RDAX-2, 1,1.5, and 2 are the NaIO_4_/xylan molar ratios, as xylan is oxidized by NaIO_4_.

Recently, a new process for the preparation of adhesives using xylan separated from PHL has been reported [79]. In this method, the xylan was oxidized using sodium periodate to generate dialdehyde xylan (DAX). Then, DAX was reduced using sodium borohydride to generate reduced DAX (RDAX) with extracyclic hydroxyl groups. After reduction, xylose can be used as an adhesive, and its properties can be adjusted using the content of extracted hydroxyl groups generated. It is believed that this process disrupted the rigid sugar ring structure, converting it into a flexible molecular chain. The lap shear strength between woodchips can reach approximately 30 MPa, far exceeding that of a commercial hot melt ethylene vinyl acetate adhesive. In addition, it exhibits good recycling performance and biocompatibility. This establishes a new application pathway for xylan separated from PHL. The mechanism of the xylan adhesive preparation and its properties can be seen in Figure 1.

#### 2.2.3. Furfural Production

As is well known, furfural is currently produced in industrial processes through the acid hydrolysis of pentosans in biomass materials such as corn cobs. In PHL, there are abundant pentoses present in both polysaccharide and monosaccharide forms, making it suitable for furfural production. Liu et al. found that when the concentrated PHL was directly used to produce furfural via acid catalyzation, the furfural yield was very low [80]. Even the lignin in the PHL was partly removed (more than 50%) via laccase-induced lignin polymerization, and when the conversion rate of xylose was greater than 90%, the yield of furfural was only close to 32–37% [60]. This is because furfural can react with itself and other components in PHL, such as lignin, sugars, intermediate molecules, and other species [81]. In order to reduce the lignin-involved side-chain reactions, a steam-stripping method has been exploited, and it has been demonstrated to improve the furfural yield to 41.5%, because the furfural formed tends to be extracted and separated from the reaction liquid by steam [80]. Mazer et al. used the PHL of aspen and maple chips to produce furfural with sulfuric acid as a catalyst, demonstrating that a furfural yield of 76% could be obtained by removing the furfural as soon as it was generated [82]. When PHL is used for furfural production, the reduction in the side reaction is crucial.

**Figure 2:**
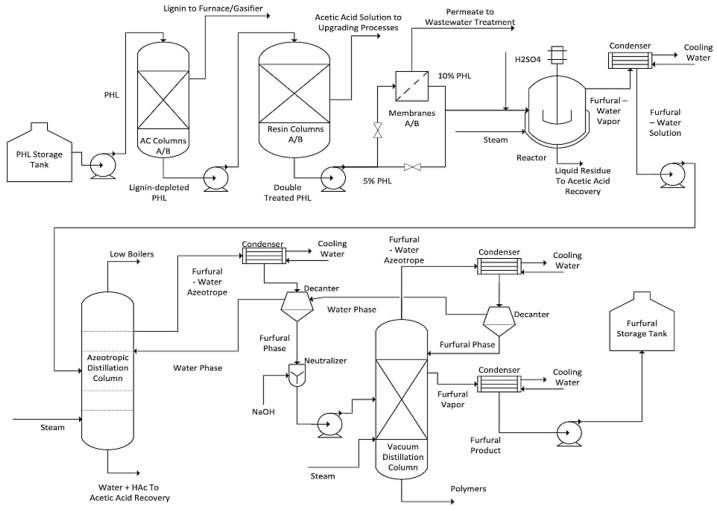
A process flow diagram for furfural production from hardwood PHL. Reprinted from [83]. Copyright (2015), with permission from Elsevier.

Another method for reducing lignin-involved side-chain reactions and improving furfural yield is to remove the lignin in the PHL before the pentoses are used to produce furfural. For example, Ni et al. exploited a process for furfural production using PHL from hardwood pre-hydrolysis as a raw material, as shown in Figure 2. In this process, activated carbon and an ion-exchange resin were used to remove and recover the lignin and acetic acid, respectively, and then the treated PHL was concentrated using a nanofiltration membrane. The concentrated PHL was heated up to 190 °C with the addition of sulfuric acid to produce furfural with a yield of 57%. In addition to participating in side-chain reactions to reduce the furfural yield, lignin can block the active sites on the ion-exchange resin and cause severe fouling on membranes, so the lignin should be removed before the PHL is used for furfural production [83].

#### 2.2.4. Production of Aldarates or Aldaric Acids

In addition to furfural, the hemicellulose sugars in the PHL can be used for the preparation of other sugar derivatives. Derrien et al. prepared aldarates and aldaric acids by oxidizing the hemicellulose sugars in softwood acid PHL with air catalyzed by Pt/C or Au−Pt/ZrO_2_. It was proven that impurities, such as lignin and furfural, could inhibit the oxidation of the sugars in PHL. After purification via a combination of filtration, an ion-exchange resin treatment, evaporation, and activated carbon treatments, hexaric and pentaric acids could be obtained by oxidizing the purified PHL, and the yields were approximately 50% of hexaric acids and 70% of pentaric acids [84]. The hexaric acids and pentaric acids could be used to produce a series of bioproducts and polymers, such as polyhydroxypolyamides and silicon polyamides [85,86].

## 3. The Utilization of the Lignin in PHL

During pre-hydrolysis, lignin is depolymerized through the cleavage of β-*O*-4 bonds and the elimination of primary and secondary aliphatic OH groups, accompanied by the formation of phenolic OH groups [87]. Compared to the technical lignin obtained from other processes, such as dioxane lignin, acetic acid lignin, and ethanol lignin, the lignin separated from PHL has a lower molecular weight and more phenolic OH groups [88]. Like other technical lignins, the lignin separated from PHL has potential applications in bioenergy, chemicals, and polymers [89].

### 3.1. Preparation of Lignin Nanoparticles

Lignin nanoparticles (LNPs) have several potential applications in the pharmaceutical and cosmeceutical sectors as well as in hybrid nanocomposites [90]. The lignin in PHL exists in the form of colloidal and dissolved lignin, which can be used for the preparation of lignin nanoparticles. Li et al. treated PHL with *p*-toluenesulfonic acid (*p*-TsOH), and the lignin tended to precipitate and was separated from the PHL. The precipitated lignin was dissolved in THF, and hollow LNPs were prepared via an antisolvent method. In addition, the remaining PHL that contains saccharides can be directly used for levulinic acid production via the catalysis of *p*-TsOH [91].

### 3.2. Lignin Absorbents

Lignin and modified lignin have been used as mesoporous biosorbents for the removal of hazardous substances from effluents, and their adsorption ability is due to functional groups, such as phenolic, hydroxyl, carboxyl, and methoxy groups, in lignin molecular structures [92]. Lignin separated from PHL can also be used as an effective adsorbent. Liu et al. treated PHL with horseradish peroxidase to separate lignin, as horseradish peroxidase can catalyze lignin polymerization which causes the lignin precipitation from the PHL. In addition, the separated lignin was rich in carboxyl groups due to the lignin oxidation catalyzed by horseradish peroxidase. It was established that the lignin absorbent’s maximum methylene blue adsorption capacity reached 241.1 mg/g at 308 K [93].

### 3.3. Preparation of Lignin-Based Carbon Quantum Dots

Gao et al. reported a process for the preparation of lignin-based carbon quantum dots via the use of PHL lignin as a carbon source. In this process, the lignin was separated from the PHL via TiO_2_ nanosheet adsorption, and then the mixture of lignin and TiO_2_ nanosheets was calcined with argon gas to obtain the lignin-based carbon quantum dots. The results demonstrated that these lignin-based carbon quantum dots improved both the separation efficiency of the photogenerated carrier and the CO_2_ adsorption capacity of TiO_2_ [94].

Compared to the lignin separated from the black liquor of the conventional alkaline pulping process, the amount of lignin separated from the PHL is much less, and its application has not been widely studied. With the development of applied biomass resources, more pathways will be exploited in the future toward the utilization of lignin separated from PHL.

## 4. Direct Utilization of PHL Without Separation and Purification

### 4.1. Preparation of Microspheres or Carbon Microspheres

PHL mainly contains hemicellulose sugars and lignin, so it can be used as a carbon source for the production of related products. For example, carbon microspheres were prepared via hydrothermal treatment of PHL at 180 °C in the presence of sulfuric acid. The prepared carbon microspheres exhibited a good adsorption capacity for Pb^2+^ and methylene blue after activation with sodium hydroxide. It was believed that the formation of carbon microspheres was mainly related to the re-polymerization or condensation of substances generated by xylose after acid hydrolysis, and the presence of a small amount of lignin in the PHL was beneficial for the formation of the carbon microspheres [9].

In an earlier paper, Edlund et al. proposed a water-in-oil emulsion technique for the preparation of microspheres using the PHL from hardwood and softwood, respectively. It was found that, in addition to the preparation factors, the hemicellulose structures and molecular weights significantly affected the viability of the PHL to form well-defined microspheres. It was believed that the prepared microspheres could be used in agriculture, for example, as easily removable spherical templates and for temporary encapsulation, etc. [95].

### 4.2. Preparation of Activated Carbon

The hemicellulose sugars and lignin in PHL are excellent carbon sources that can be used to produce activated carbon. Cao et al. activated the PHL of poplar wood by adding KOH and prepared porous carbon with a high specific surface area via calcination in the presence of N_2_; the adsorption capacity for methylene blue was up to 680 mg/g [96]. However, the solid content in the PHL was relatively low, the heat consumption for evaporation was high when using PHL to prepare porous carbon, and the economic viability was an issue.

### 4.3. Preparation of Biomass Films

**Figure 3:**
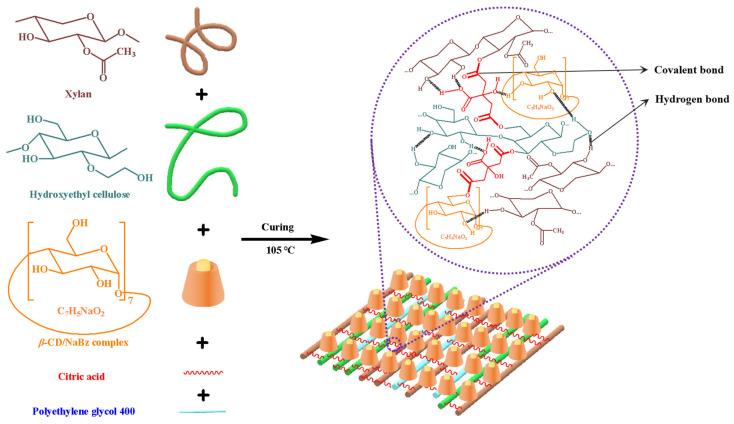
The forming pathway of the antimicrobial film based on xylan and HEC with CA as a crosslinker. Reprinted from [99]. Copyright (2019), with permission from Elsevier.

As natural polymers, hemicelluloses can be used for film preparation in the food industry. In general, compared to other biopolymers, the hemicellulose of lignocellulose is not suitable for film preparation due to its weak mechanical strength and high hydrophilicity [97,98]. In addition, during acidic pre-hydrolysis, hemicelluloses tend to be degraded into hemicellulose sugars with lower molecular weights. In order to improve the performance of the hemicellulose-based film, such as the mechanical properties and oxygen permeability, a modification of the hemicelluloses or an enhancement via the addition of other polymers is needed. For example, the xylan precipitated from PHL by ethanol and hydroxyethyl cellulose (HEC) was used to prepare composite films, with citric acid (CA) as a crosslinker and polyethylene glycol as a plasticizer, and the introduction of the β-cyclodextrin/sodium benzoate (β-CD/NaBz) complex into the composite matrix improved the antimicrobial activity of the composite films [99]. The composite film’s formation pathway can be seen in Figure 3.

**Figure 4:**
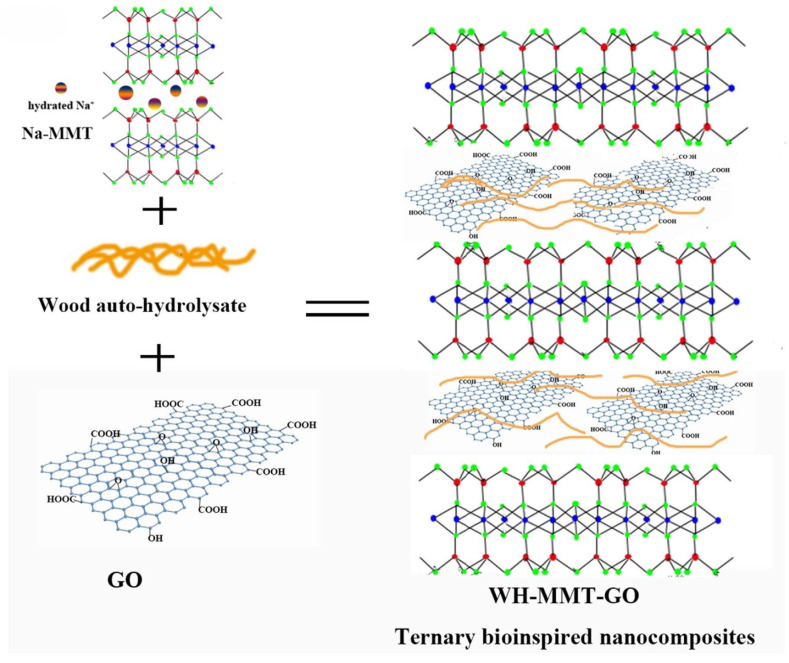
The forming mechanism of ternary nanocomposite films. Reprinted from [100]. Copyright (2018), with permission from Elsevier.

**Figure 5:**
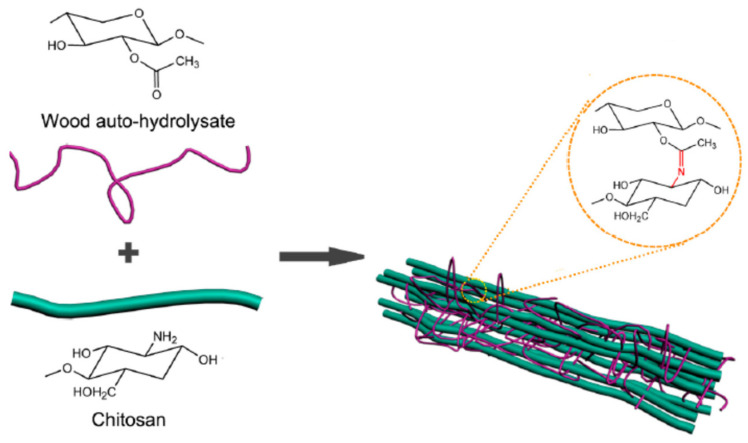
The forming pathway of composite films based on wood auto-hydrolysates (WAH) and chitosan (CS). Reprinted from [101].

In addition to the use of PHL-separated xylan for film preparation, hemicellulose-containing PHL can also be used for the direct preparation of biofilms without separation. Chen et al. prepared a bioinspired nanocomposite film via the combination of PHL with montmorillonite (MMT) and a small quantity of graphene oxide (GO). The prepared films had high strength values and fire resistance properties, and the introduction of GO endowed the films with hydrophobic properties, which made the PHL useful for the fabrication of fire-resistant films, coatings, and packaging [100]. The forming mechanism of ternary nanocomposite films can be seen in Figure 4. Other polymers with excellent film-forming properties, such as chitosan (CS), can be added into wood auto-Hydrolysate (WAH) for composite film preparation, and crosslinking interactions between WAH and CS are due to Millard reactions. It was found that the WAH/CS ratio benefits the tensile strength, light transmittance, and thermal stability of PHL-based composite films, and the addition of CS resulted in a decrease in the oxygen transfer rate and water vapor permeability of the films [101]. The forming pathway of WAH/CS composite films can be seen in Figure 5.

In addition to chitosan, carboxymethyl cellulose (CMC) is also a common polymer used to improve the hemicellulose-containing PHL-based films’ performance [102,103]. Ryberg et al. found that a filtration step for the removal of small-molecular-weight compounds such as furfural was required for PHL-based film preparation, especially for potential food packaging applications, and the presence of lignin in the films was beneficial for the oxygen barrier performance at higher humidities [103].

The advantage of preparing biomass films directly using hemicellulose-containing PHL is the exemption of complex separation steps; in addition, the lignin in PHL can endow special properties, such as UV resistance and antibacterial effects, on the films. However, monosaccharides and other impurities in PHL, such as acetic acid and furfural, may negatively impact the performance of the films.

## 5. Recovery of Acetic Acid from PHL

**Figure 6:**
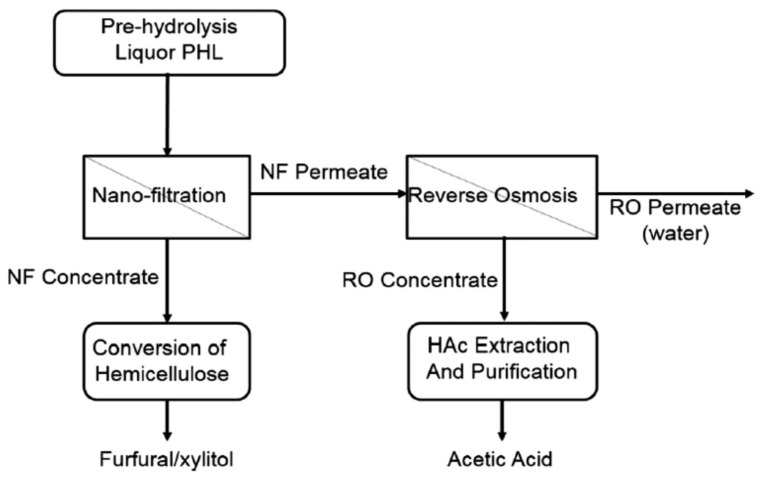
Process flow diagram for biorefinery. Reprinted from [106]. Copyright (2015), with permission from Elsevier.

The main hemicellulose in hardwoods and softwoods are *O*-Acetyl-4-*O*-methyl-glucurono-xylan and *O*-Acetyl-galactoglucomannan, respectively [4,5]. During the pre-hydrolysis of lignocelluloses, the acetyl groups in hemicelluloses can be hydrolyzed in acidic conditions to generate acetic acid, which can catalyze further hydrolysis reactions [104]. Acetic acid can be recovered as a valuable chemical. In general, the acetic acid concentration is low, but with the recycling of the PHL used for hydrolysis, the acetic concentration in PHL increases substantially [105]. Due to the low concentration of acetic acid in PHL, it is not cost-effective to recover it separately, but it can be retrieved while recovering hemicellulose sugars. Ni et al. analyzed a process for the recovery of hemicelluloses and acetic acid, and the process flow diagram is shown in Figure 6. In this process, nanofiltration (NF) was carried out to separate the hemicelluloses in the original PHL. The hemicellulose in the NF concentrate section was used for the production of furfural or xylitol, and the NF permeate section was concentrated via reverse osmosis (RO), and then the RO concentrated section was extracted with tertiary amines and polar organic diluents to separate acetic aciD (HAC) [106].

Ni et al. also proposed another process for acetic acid recovery, in which the PHL is extracted with triisooctylamine (TIOA) diluted with decanol after being treated with activated carbon for lignin removal. It was found that the TIOA dilution, temperature, and pH significantly affected the acetic acid recovery efficiency [107].

The acetic acid in PHL can also be recovered via adsorption by ion-exchange resins. To prevent lignin in the PHL from blocking the active sites on the ion-exchange resin, the prior resin was used for acetic acid adsorption, and activated carbon was used to remove the lignin in the PHL. It was demonstrated that, compared to the quaternary amine-based resin, the tertiary amine-based resins performed better in regard to the acetic acid adsorption efficiency. In addition, the acetic acid on resins could be desorbed via elution using a sodium hydroxide solution [108].

## 6. Conclusions

(1) The efficient utilization of various biomass components in PHL is of great significance for the complete utilization of biomass resources. The sugars, lignin, and acetic acid components in PHL can be effectively separated and utilized accordingly. The exploitation of efficient separation technologies is particularly important for the high-value utilization of each component in order to reduce the loss of target components.

(2) Many methods have been exploited for the separation and purification of hemicellulose sugars from PHL; a reasonable combination of different methods can achieve greater impurity removal and less sugar loss. Different adsorbents, such as lime, activated carbon, ion-exchange resin, or their combination, are currently being applied in industrial production to separate and purify hemicellulose sugars. During adsorption, the adsorbent type, dosage, and treatment conditions significantly affect the efficiency of the impurity removal and the hemicellulose sugar yield.

(3) The hemicellulose sugars from PHL can be used in conventional fields such as the food and pharmaceutical industries, but their other applications as polymers should be further explored, and the properties and costs should also be considered.

## 7. Challenges and Prospects

(1) The most popular and feasible method for separating PHL components employs adsorbents; however, these methods still have high usage rates, resulting in additional waste or excessive sugar loss. In addition, adsorbed components such as lignin are difficult to recover. For adsorption processes for the separation of biomass components, the adsorbents should be easily recycled, and no new purifies or additional waste should be introduced. Furthermore, the utilization of the adsorbed components, such as lignin, should be considered.

(2) The utilization of PHL without separation is an alternative process for the full utilization of PHL components. In general, for biofuel production through the fermentation of hemicellulose-based PHLs, the detoxification achieved by removing inhibitors, mainly lignin, in PHL is important for improving the fermentability of the PHL. When PHL is used for biofuel production without separation, it is essential to optimize the microorganisms that are insensitive to inhibitors, such as lignin and furfural.

(3) The relatively lower molecular weight of the hemicelluloses and the presence of some low-molecular-weight components, such as acetic acid and furfural, in PHL are disadvantageous for their application in the fields of materials or polymers. The primary separation of PHL to remove low-molecular-weight substances and the classification of hemicelluloses via membrane filtration are beneficial for its utilization as a hemicellulose-based material and to improve the properties of the target products.

(4) In addition to hemicelluloses, the lignin in PHL also has broad potential applications worth exploring. During the separation, the simultaneous modification and separation of the lignin will be beneficial for its further utilization.

## Figures and Tables

**Table 1:** Comparation of different methods for impurities removal from PHL.

Raw Materials for Pre-Hydrolysis	Component Concentration of Original PHL(g/L): Total Sugar/Lignin//Furfural/5-HMF/Acetic Acid	Treatment Methods	Impurities Removal/%				Sugars Loss/%	Reference
			Lignin or Phenolic Compounds	Furfural	5-HMF	Acetic Acid		
Spruce wood chips	15.6–26.6/10.7–14.3/0.32–4.50/NA/0.89–1.32	Acidification (H_2_SO_4_)	0–6.8	36.8–76.7	NA	42.7–82.4	0–9.9	[22]
Sugar maple chips	37.91/5.55/1.34/0.33/7.24	PDADMAC coagulation	52.7	67.9	57.6	38.2	36.8	[68]
Mixture of Maple, poplar and birch	7.20 and 50.33/5.35 and 9.22/1.43 and 1.53/NA/9.23 and 10.11/	CaCO_3_ adsorption	15.0 and 9.8	0 and 9.1	NA	14.2 and 17.3	5.4 and 0	[13]
Mixture of Maple, poplar and birch	24.9/28.6/2.0/NA/NA	Acidification + Ca(OH)_2_ treatment	55	11	NA	NA	11	[25]
Poplar wood chips	45.0/17.9/NA/NA/3.5	Ion-exchange resin ((IRA-400)(OH^−^)) adsorption	79.5	NA	NA	43.9	9.5	[48]
Mixed hardwood	114.4 ^a^/42.9/7.8/1.9/NA	Polystyrene divinylbenzene (PS-DVB) resin adsorption	95.1	92.3	97.9	NA	4.0	[40]
Mixture of Maple, poplar and birch	24.9/28.6/2.0/NA/NA	Acidification + Ca(OH)_2_ treatment + Two stages of AC adsorption	76.2	85	NA	NA	18.9	[69]
Eucalyptus wood chips	40.6/9.7/1.9/0.4/6.2	Ca(OH)_2_ treatment + AC adsorption + Laccase treatment + AC adsorption	90.4	100	NA	NA	9.9	[8]
Poplar wood chips	11.83/5.07/0.63/NA/1.55	Ca(OH)_2_ + AC adsorption	66.9	70.1	NA	NA	5.9	[32]
Eucalyptus sawdust	15.79 ^b^/5.33/0.25/0.073/1.51	Ethyl acetate extraction	63–82	92	66	NA	15	[58]
Mixture of Maple, poplar and birch	61.9/12.2/NA/NA/11.3	Laccase treatment + cationic polymer flocculation	46–61	NA	NA	37.4–42.9	12–15	[70]
Mixture of Maple, poplar and birch	50.33/9.22/1.43/NA/10.11	AC adsorption + ion-exchange resin (Purolite A103S)	80	70	NA	70	12	[67]

^a^ After acid hydrolysis of original PHl; ^b^ xylo-oligosaccharides.

## Data Availability

No new data were created or analyzed in this study. Data sharing is not applicable to this article.
